# A multiscale X-ray CT study of degradation and safety behaviour of different Li-ion battery positive electrode morphologies

**DOI:** 10.1039/d6eb00099a

**Published:** 2026-09-15

**Authors:** Hamish T. Reid, Drasti Patel, Will J. Dawson, James B. Robinson, Rhodri Jervis, Paul R. Shearing

**Affiliations:** a Electrochemical Innovation Lab (EIL), Department of Chemical Engineering, University College London (UCL) London WC1E 7JE UK paul.shearing@eng.ox.ac.uk; b Advanced Propulsion Lab (APL), Marshgate, UCL London E20 2AE UK; c The Faraday Institution, Harwell Science and Innovation Campus Didcot OX11 0RA UK; d The ZERO Institute University of Oxford, Holywell House, Osney Mead Oxford OX2 0ES UK

## Abstract

While battery thermal failures are rare, improving our understanding of battery state-of-safety is essential to further reduce failure rates and support the energy transition. Although batteries undergo rigorous certification, the evolution of safety characteristics with cell ageing remains poorly understood. Here, electrochemical testing accelerating rate calorimetry (ARC), and multiscale X-ray computed tomography (CT) are combined to investigate how state-of-safety of lithium-ion pouch cells containing single-crystal or polycrystalline NMC cathode evolves with cycling. Comparable pouch cells were assessed before and after cycling to 80% capacity retention. The single-crystal containing cells gave an approximate 50% improvement in cycle life compared with polycrystalline cells. In the pristine state, ARC showed that the single-crystal cells had a lower self-heating onset temperature but also a lower peak temperature than polycrystalline cells, indicating lower thermal stability but less destructive failure. After cycling, the single-crystal cells showed higher self-heating onset temperature and lower peak temperatures. In contrast, the polycrystalline cells showed reduced self-heating onset temperatures and thermal runaway initiation temperatures indicating a loss of thermal stability. Multiscale X-ray CT provided mechanistic insight into these changes by linking safety behaviour to degradation across multiple length scales. Whole-cell imaging revealed greater gas generation, electrode displacement, and loss of layer contact in the polycrystalline cells, while high-resolution CT identified extensive particle cracking and fragmentation that was largely absent in the single-crystal material. These observations suggest that ageing-induced particle fracture increases the reactive cathode-electrolyte interfacial area, accelerating exothermic reactions during thermal abuse and reducing thermal stability. This work demonstrates how multiscale X-ray CT can reveal the mechanisms that influence the changes in of battery state-of-safety with cycling and highlights the potential safety advantages of single-crystal cathode architectures in aged lithium-ion batteries.

## Abstract 2

Electrochemical methods, accelerating rate calorimetry, and multiscale X-ray computed tomography are combined to investigate how state-of-safety of lithium-ion pouch cells containing single-crystal or polycrystalline NMC cathode evolves with cycling.

## Graphical abstract

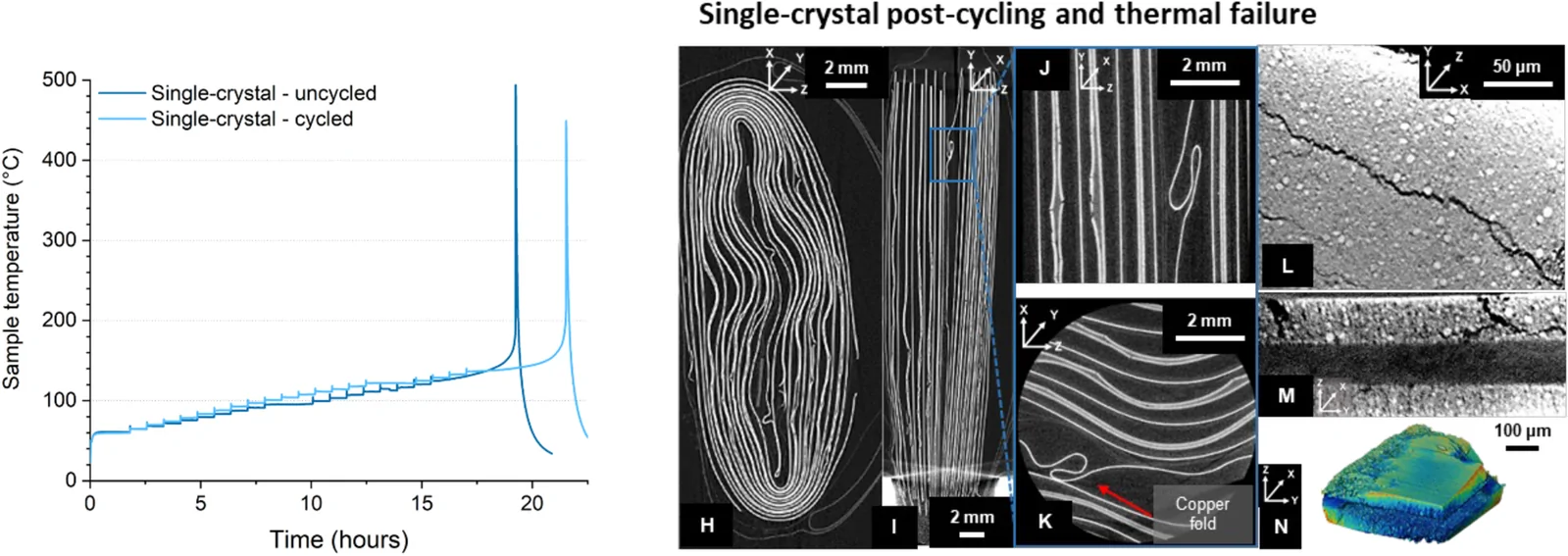

**Broader context**

Whilst battery thermal failures are rare, there is a need to improve our understanding of the Science of Battery Safety, to further reduce failure rates and ensure economic and equitable energy storage to support the energy transition. Whilst, in general, new batteries are subjected to robust qualification and certification protocols, our understanding of how battery state-of-safety evolves with lifetime and associated cell degradation remains limited. This is particularly important for cells in demanding duty cycle applications, where there may be substantial arising heterogeneities. Here we apply a portfolio of techniques to evaluate how the safety characteristics of different cathode architectures evolves over extended lifetime operation, paving the way for a more robust definition of battery state-of-safety in real world scenarios.

## Introduction

Battery research efforts over the previous 20 years have focussed heavily on increasing energy density, power capability and cell lifetime.^1–3^ However, as the performance of batteries increases, concerns have been raised about the safety behaviour of such devices, with some high-profile battery failures leading to fires and thermal runaway.^4,5^ Although these failures are rare, thermal stability and overheating may pose a significant risk to devices and users. The stability of batteries is known to be affected by external conditions; for example, improper storage and manufacturing defects can make cells unsafe. Although the safety behaviour of many cells and their chemistries is well understood in the pristine state, through widespread certification standards, the underpinning impact of long-term charge/discharge cycling is still unclear. As stockpiles of used batteries grow there is an imperative for effective second and third life solutions, and, consequently, the correlation between ageing and safety has recently gained more attention.^6^

Carter *et al.* investigated the effects of cycling at 0 °C *versus* ambient temperature in cylindrical cells with and without a mandrel, using X-ray computed tomography to image the interior of the cell before teardown analysis.^7^ This work showed how the lack of a mandrel, a thin cylinder of plastic or metal used to wrap the jellyroll around, led to collapse of the electrode layers into the core of the cell, while lithium plating caused a loss of thermal stability after cycling. Similar behaviour in cold-cycled cells has also been observed in other studies using graphite anodes and layered oxide positive electrode (cathode) materials.^8–10^ Lui *et al.* used an overcharging procedure to investigate the safety characteristics of commercial NMC-graphite cells at different states-of-health (SOHs). The authors found the thermal stability of the cells was affected by reactions on both the negative electrode (anode) and cathode sides.^11^ Reviews from Preger *et al.* and Wang *et al.* provide comprehensive summaries of many studies that have investigated the links between battery cycle ageing and safety.^6,12^

To improve the cycle life of layered oxide cathodes, single-crystal active materials have received recent research attention.^13–16^ Single crystals lack the secondary particle architecture of their polycrystalline counterparts. This renders the particles more resistant to anisotropic strain during charge and discharge, preventing particle cracking and capacity loss.^17,18^ Recently, Liu *et al.* used a conductive network of Li_1.3_Al_0.1_Sc_0.2_Ti_1.7_(PO_4_)_3_ to further improve the cycling performance at high cutoff voltages.^19^ However, the difference in microstructure between the two crystal formats could give rise to differences in the safety performance as well. For example, single-crystal materials generally have a greater specific surface area in the pristine state, increasing the number of active sites where reactions between the cathode and electrolyte could be initiated.^20–23^ So far, single component studies have suggested that single-crystal materials are more thermally stable than polycrystalline, based on oxygen release between the grain boundaries in polycrystalline materials.^24,25^ However, these works have generally considered individual components rather than exploring thermal stability in the whole-cell system.

X-ray computed tomography has been used extensively for the non-destructive characterisation and visualisation of batteries and battery materials at multiple length scales.^26,27^ Both high-speed X-ray radiography and X-ray computed tomography have been successfully employed to observe changes in battery structure during capacity degradation and thermal runaway. Examples of X-ray CT applied to battery failure include Wu *et al.*, where the authors applied whole-cell CT to failed cells of different formats.^28^ The sequence of events that occurred in the cell leading up to and during thermal runaway were evidenced for both cylindrical and pouch cells. Finegan *et al.* used both synchrotron and lab-based X-ray sources for *operando* imaging during overcharge-induced failure, and post-mortem tomography of the cathode particle to observe changes in the microstructure.^20^

Here, a multi-scale X-ray CT approach was employed to compare the cycling and safety behaviour of single-crystal and polycrystalline materials.^29^ Each cell delivered the same nominal capacity and contained the same graphite anode and organic electrolyte. First, a heat-wait-search (HWS) calorimetry protocol was used to evaluate the different safety performances between the different cathode morphologies in the pristine state. Calorimetry was used to determine the characteristic temperatures in the lead up to thermal runaway: onset of self-heating, initiation of thermal runaway and peak temperature. Cells were also cycled to 80% capacity retention to evaluate the electrochemical differences between the two morphologies, with post-cycling CT used to investigate the changes that had occurred on both the cell and particle level during cycling. Cycled cells then underwent the same HWS protocol to understand how cycle ageing had affected the safety performance, with post-mortem CT again used to probe the failed cells at various length scales. Finally, scanning electron microscopy (SEM) is used to qualitatively evaluate the single-crystal particle in cycled and failed conditions. Through this methodology, it is hoped that this work will contribute to the understanding of battery safety after ageing, and the effect of active material microstructure on thermal stability.

## Experimental

### Pouch cell preparation

402035-size pouch cells were obtained dry (no electrolyte) from Li-Fun Technology (Li-Fun technology, China). The cells consisted of polycrystalline and single-crystal NMC811 positive electrode material coated onto aluminium with a loading of 15.52 mg cm^−2^ and 12.26 mg cm^−2^ respectively. The formulations for the positive coating contained 96.4% and 95.5% active material (AM) for the polycrystalline and single-crystal coatings respectively. The negative electrode material for both sets of cells was artificial graphite coated on to copper, with a loading of 11.28 mg cm^−2^ and 8.75 mg cm^−2^ (both 95.7% AM) for the polycrystalline and single-crystal cells respectively. Cells were opened and dried in a vacuum oven at 100 °C overnight to remove any residual water, before filling in an argon-filled glovebox with 1.0 M LiPF_6_ in 3 : 7 EC/EMC electrolyte. The cells were clamped in holders to provide pressure (approx. 0.8 MPa) and formed using 10 mA (C/20) for two cycles between 2.5–4.2 V using a Biologic BCS-815 battery cycler (Biologic, France). Once formed the cells were degassed and resealed in an argon-filled glovebox.

### Pouch cell cycling

Charge/discharge cycling was performed at 100 mA (C/2) using a Biologic BCS-815 cycler. Every 50 cycles, two ‘diagnostic’ cycles were performed at C/20 to obtain differential capacity curves. The cells were secured in a spring-loaded clamped holder during cycling to apply even pressure. The cells were kept in a temperature-controlled chamber at 25 °C for the duration of cycling. Cells that were later used for calorimetry studies were cycled until they reached 80% of their discharge capacity recorded in the first cycle. The cells represented in Fig. 2 were left to cycle beyond 80% capacity fade to better understand the whole capacity profile.

### Accelerating rate calorimetry

**Fig. 1:**
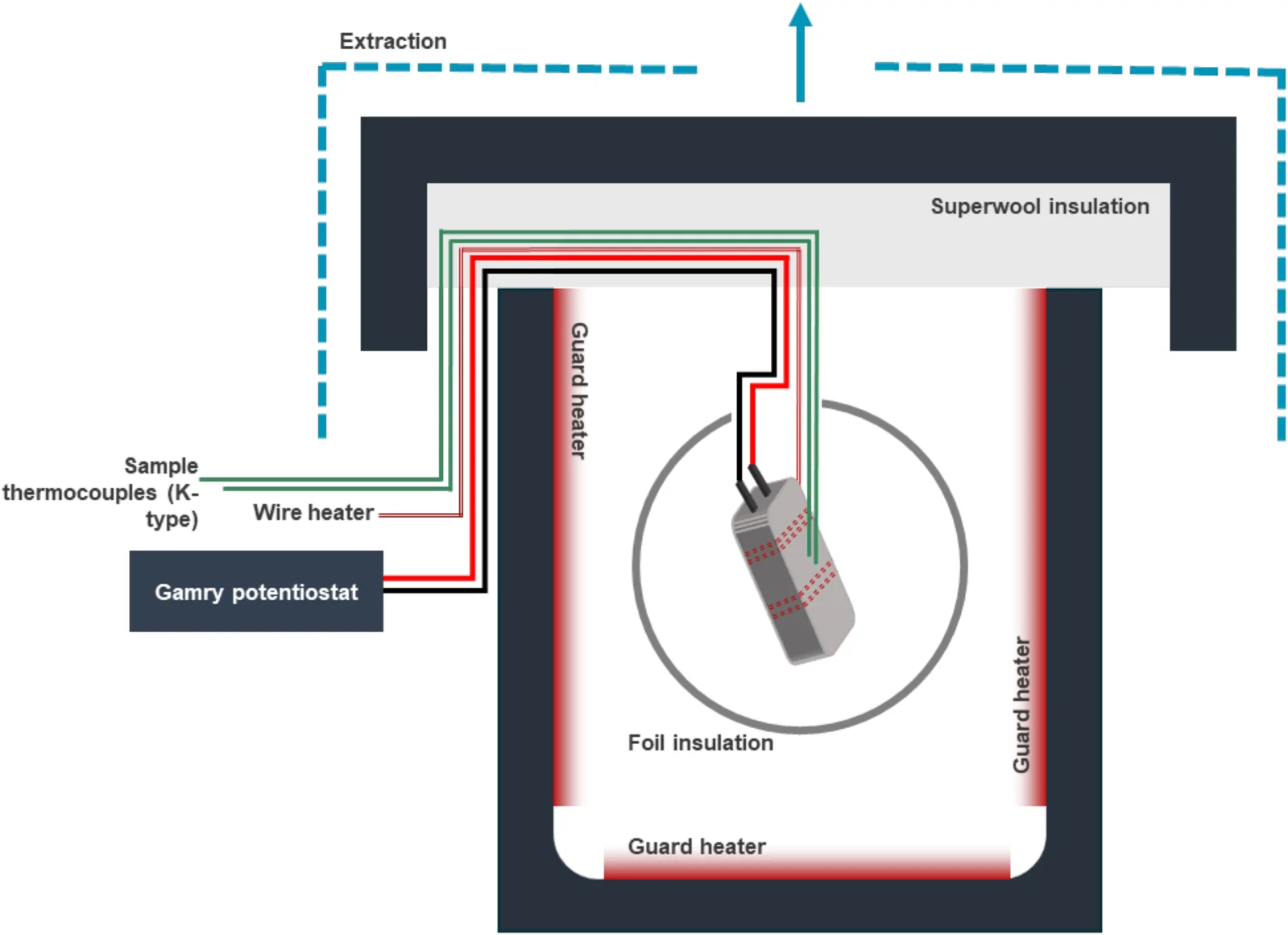
Schematic of the calorimeter set up used in this work.

A HEL BTC-130 (HEL Group, UK) calorimeter was used for all destructive testing. A HWS protocol was used to identify specific temperatures during cell failure. The starting temperature used was 50 °C, and then the temperature was increased by 5 °C increments. A wait time of 15 minutes was used for each step, after which the instrument would search for exothermic reactions with a detection limit of 0.02 °C min^−1^. Once a temperature increase was detected, the calorimeter goes into to tracking mode, matching the calorimeter vessel temperature to that of the cell to maintain an adiabatic environment. Two Type-K thermocouples were attached near the centre of cell, one connected to the calorimeter and other connected to a Picolog TC-08 data logger (Pico Technology, UK), to ensure reliable temperature measurements. An insulated Ni-chrome heater wire was wrapped around the cell for even and effective heating. The full cell, thermocouple, and heater wire assembly was placed inside the centre of the calorimeter, as shown in Fig. 1. Each cell, with thermocouples and heater wire taped on, was wrapped loosely in aluminium tape to dissipate radiant heating from the guard heaters and prevent any hot spots. A Gamry Interface 1010 E potentiostat (Gamry Instruments, USA) was used to charge the cells to 4.2 V using a constant current of 100 mA (C/2) and apply a constant voltage hold at 4.2 V for three hours once they were in the calorimeter, and monitored the voltage during the HWS test.

### Multi-scale lab-based X-ray computed tomography

Full cell scans were performed using a Nikon XT 225 (Nikon Metrology, UK) X-ray CT system. A tungsten source and accelerating voltage of 180 kV were used to take 3185 projections and achieve a voxel resolution of *circa*. 16 μm. Datasets were reconstructed using CT Pro 3D software.

Higher magnification scans were performed using a Zeiss Xradia 620 Versa (Carl Zeiss AG, Germany), using an accelerating voltage of 80 kV with a tungsten source. Images were taken of the cell in the pristine, aged and post-failure state using 0.4× and 4× objective lenses. Cells were then dismantled after thermal runaway and the cathode material was removed from the centre of the cell. A ‘tab’ geometry was used, where the scanned area measured 0.4 mm × 0.4 mm to match the field-of-view of the instrument.^30^ The main tab body measured 4 mm × 1.5 mm and was mounted on to a dressmaker's pin using epoxy. A 40× lens was used on the same CT instrument to acquire images of the cathode particles. Table 1 shows the parameters used to acquire images at the different length scales used in this work. Zeiss Reconstructor software suite was used to reconstruct the higher magnification (40×) scans, and the Optirecon package was employed for the post-thermal runaway materials (4× and 0.4×). All reconstructed datasets were visualised and analysed using Avizo 3D software (Thermo Fisher Scientific, USA).

**Table 1:** Listing of the different imaging instruments and parameters used in this study

Instrument	Objective lens	FOV/mm	Voxel size/μm
Nikon XT H225	NA	30.0	15.7
Zeiss 620 Versa	4×	3.70	1.8
	40×	0.380	0.2

Particle size distributions were calculated from the 40× micro-CT datasets, using samples taken from near the centre of each cell. Each dataset was filtered using a non-local means algorithm in Avizo to improve edge definition for particle identification. The resulting dataset was segmented into two phases: the spaces between particles and particles themselves using a greyscale-based thresholding technique. A separation algorithm was used to distinguish the individual particles from each other, where each could be labelled individually. Any particles that were on edges of the image, where only a portion were visible, were removed to reduce biasing towards smaller particle sizes. The particle size distribution calculation used approximately 8000 particles from each dataset, taken within a FOV of approximately 0.4 × 0.4 mm and across the whole thickness of a single coating of electrode. Only one dataset was used for each cell condition, as recovering material from the post-thermal runaway cells proved challenging.

### Scanning electron microscopy

SEM images were acquired using a Zeiss EVO 10 SEM (Carl Zeiss AG, Germany). The SE1 signal was used with an accelerating voltage of 15.0 kV to capture images at 1250×, 2500× and 5000× magnification. The pixel sizes were approximately 235, 126, 57 nm respectively. Samples extracted in an argon-filled glovebox. The samples were attached to separate steel SEM stubs using carbon tape, before transfer to the SEM in a sealed container.

## Results and discussion

### Electrochemical comparison between polycrystalline and single-crystal cells

**Fig. 2:**
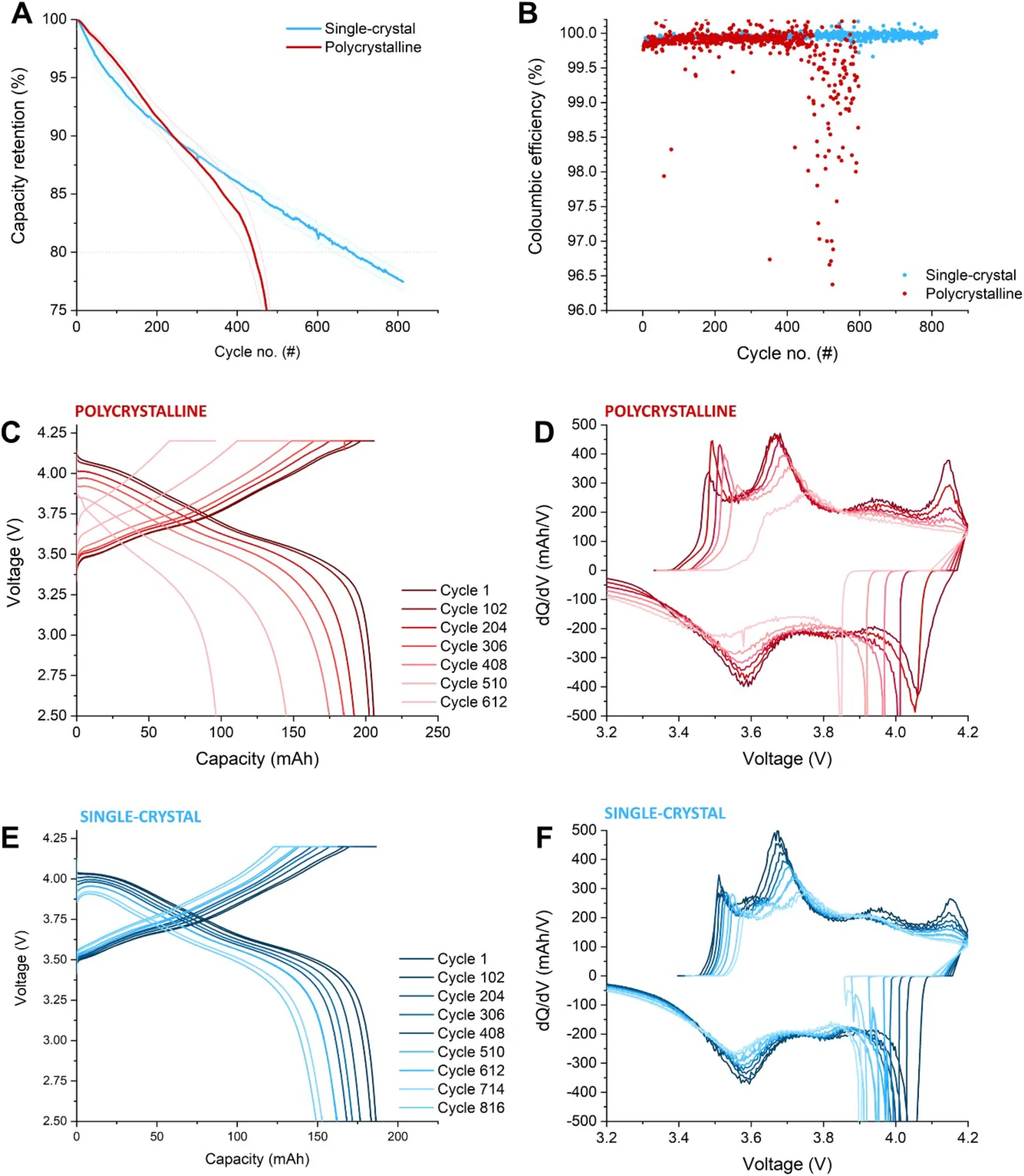
Electrochemical cycling data from representative polycrystalline and single-crystal cells. (A) Discharge capacity retention per cycle, with 100% capacity the amount delivered in the first cycle. Highlighted area around the capacity curve indicates the represents the average percentage standard deviation error. (B) Coulombic efficiency values for the same cells as in (A). (C and E) Voltage *versus* capacity curves, taken every 100 cycles for polycrystalline (red, top) and single-crystal (blue, bottom) cells respectively. Darker colours represent earlier cycles (D and F) d*Q*/d*V* analysis plots, taken every 100 cycles, for polycrystalline (red, top) and single-crystal cells (blue, bottom) respectively. Darker colour represents earlier cycles.

The discharge capacities for representative cells of both types that were cycled until they reached greater than 20% capacity fade are shown in Fig. 2A. Similarly, the differential capacity analysis (DCA) and capacity *versus* voltage plots are shown in Fig. 2B–E. Under the present conditions, the single-crystal cells show improved long-term cycle life, taking 225 more cycles to reach 80% capacity retention. These results broadly agree with the findings of Lüther *et al.*, who also found that single-crystal materials give improved cycle life in high-nickel cathodes, although at the cost of specific capacity.^15^ The polycrystalline cells showed steady capacity fade at the beginning of cycling, with the rate of degradation increasing rapidly after 445 cycles. The knee-point in the cycling profile, where the rate of degradation suddenly accelerated, is associated with several degradation modes, one of which is particle cracking.^31^ Active particles eventually reach a point where the strain of repeated cycles has become sufficient to cause irreversible cracking. The cracking causes the loss of electronic conductivity through a reduction in connectivity between particles. Fracturing also leads to the exposure of the fresh cathode surface, which leads to swift loss of capacity. On the other hand, the single-crystal cathode materials exhibit more uniform capacity loss throughout the cycling process, suggesting that degradation modes other than cathode particle cracking are the cause of irreversible capacity fade. For example, Zhang *et al.* recently highlighted oxygen migration, and subsequent atomic restructuring, as a key degradation in high-nickel single-crystal cathodes.^32^

The coulombic efficiencies (CEs) of the two material types are also shown in Fig. 2B. The single-crystal cells show very close to 100% coulombic efficiency throughout 800 cycles, whereas the CE of the polycrystalline cells remained stable until the capacity ‘knee-point’ around 445 cycles, where the CE becomes erratic and begins to drop below 99–98%. The initial steady capacity fade of both sets of cells is likely due to anode-side degradation. As has been discussed by Dose *et al.*, anode slippage may force the cathode voltage higher with repeated cycles, eventually leading to the drop in capacity and efficiency seen after the knee-point.^33^ Because the sharp drop in capacity is observed here in polycrystalline cells and not in the single-crystal based cells, this could be due to particle fragmentation. Alternatively, cross talk between the anode and cathode sides could also be responsible, where degradation products on the cathode accelerate anode-side degradation.

The voltage-capacity plots for both materials also reflect the degradation seen in the capacity plot. The voltage drop at the start of discharging of the polycrystalline cells in Fig. 2C also accelerated with extended cycling, whereas the single-crystal cell (Fig. 2E) showed a more gradual decrease, and similar patterns were observed for increasing initial voltage during charge. It was also observed that the single-crystal cells show a small increase in voltage at the start of discharge, which increases with successive cycling, visible in Fig. 2E between 0–25 mAh, which was not seen in the polycrystalline cells. During Cycles 1 and 102 (diagnostic cycles) there was no major increase in voltage at the start of discharging recorded in the single-crystal cell. However, by Cycle 612 this increase starts to become apparent, whereby at Cycle 816 the voltage increased by approximately 5 mV at the beginning of discharge. As this effect is seen only in the later cycles of the single-crystal cells, and not at all in the polycrystalline, it may be due to degradation on the anode side leading to a reduction in ion mobility, possibly due to growth of the SEI layer.^34,35^ As the SEI grows thicker, the impedance associated with lithium transport across the anode-electrolyte interface increases, requiring a greater overpotential before discharge proceeds. This results in the progressively more pronounced feature at the start of discharge shown in Fig. 2E.

The DCA plots (Fig. 2D and F) show effects of both voltage and capacity fade. As expected, the major peaks were observed at the same voltages for both morphologies as they were both the same cathode and anode chemistry. However, the polycrystalline cells showed an accelerated decrease in the magnitude of the major peaks, as well as a shifting towards higher voltages during charge and lower voltage during discharge, implying voltage fade as well as capacity fade. For example, the second peak, around 3.6–3.7 V, is typically attributed to H1 **→** M phase transition. From the polycrystalline DCA results in Fig. 2D, the H1 → M peak reduced in magnitude by 50%, from 240 mAh V^−1^ at Cycle 1, to 120 mAh V^−1^ at Cycle 408, taking a baseline around 230 mAh V^−1^. In the single-crystal results shown in Fig. 2F, the same peak reduces in magnitude from 286 mAh V^−1^ at Cycle 1 to 205 mAh V^−1^ at Cycle 408, a reduction of 28%. In the single-crystal results th We we same H1 → M peak also shifts from 3.67 V at Cycle 1 to 3.69 V at Cycle 408. Whereas the polycrystalline shows a shift from 3.67 V to 3.72 V from Cycle 1 to 408. The polycrystalline material also sees a large drop in magnitude, around 25% between Cycle 408 and 510, and a shift in peak position to 3.76 V. The accelerated loss of magnitude and peak shifting in the polycrystalline cell shows that there is greater resistance in the electrodes with ageing as well as loss of capacity. The peak shift indicates it takes a higher voltage to access the capacity from the positive active material phase transitions. The change in peak magnitude indicates that less capacity is available when the transition happens.

### Calorimetry comparison between polycrystalline and single-crystal cells

**Fig. 3:**
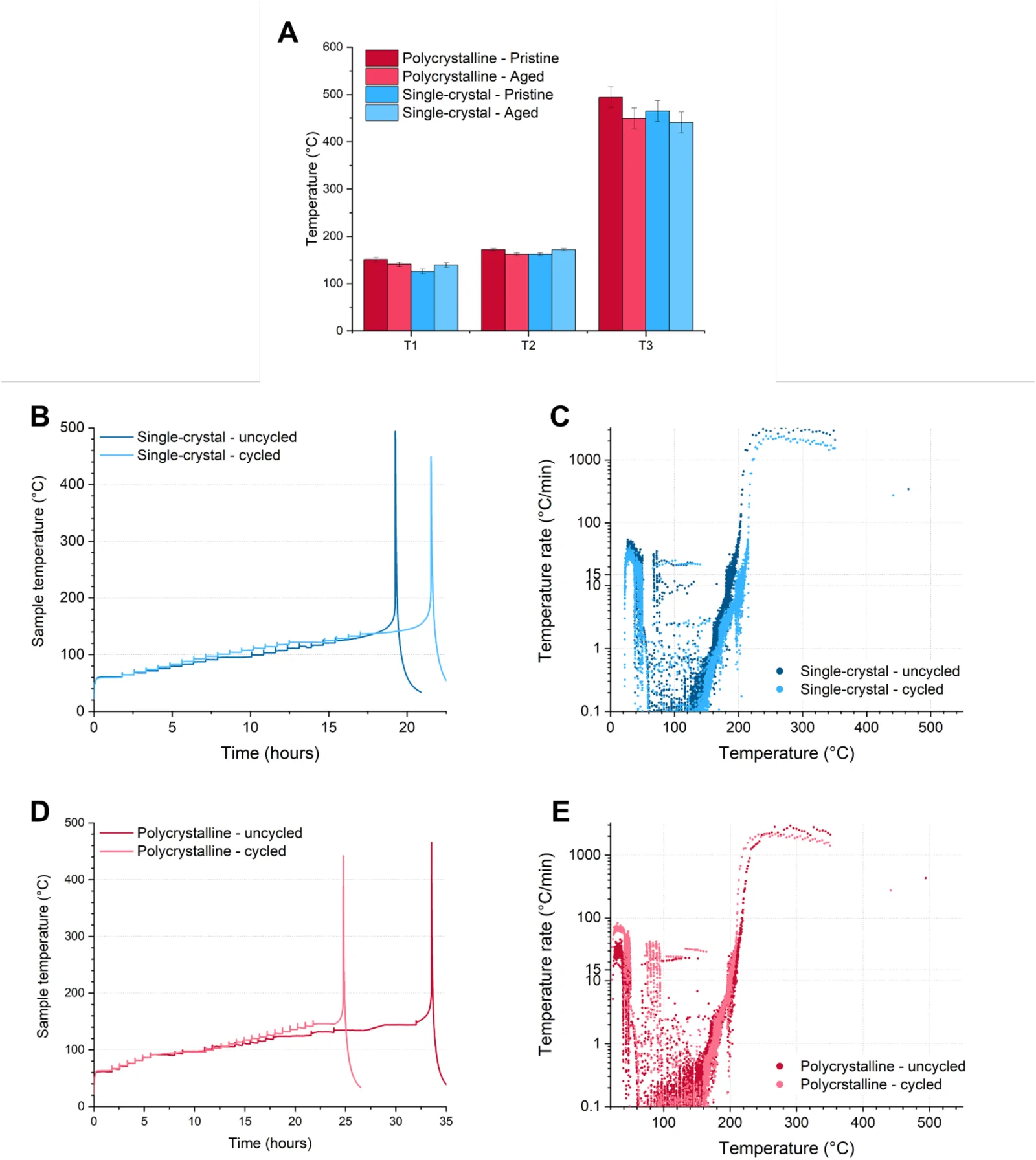
Heat-wait-search calorimetry data from polycrystalline and single-crystal cells, both cycled and uncycled. (A) top Bar graph indicating the key characteristic temperature for uncycled, cycled polycrystalline and single-crystal cells, where T_1_ = onset of self-heating, T_2_ = initiation of thermal runaway, and T_3_ = peak thermal runaway temperature. (B) Temperature over time profile for a cycled and uncycled polycrystalline representative cell. (C) Temperature over time profile for cycled and uncycled single-crystal representative cells. (D) Heat rate *versus* temperature profiles for a representative polycrystalline cell. (E) Heat rate *versus* temperature profiles for a representative polycrystalline cell.

Pristine cell of both types were filled, sealed, formed, and de-gassed before being transferred directly to the calorimeter for final charging and testing. HWS ARC was used to find the characteristic temperatures of onset of self-heating, initiation of thermal runaway and peak temperature, and therefore to assess the safety performance. Results of this testing are shown in Fig. 3. All cells were charged to 4.2 V prior to beginning the calorimetry test to simulate a “worst-case” scenario under heating at full SoC. For this work, the onset of self-heating (T_1_) was the point at which a heating rate of 0.02 °C min^−1^ was exceeded; the intitiation of thermal runaway was determined to be the point (T_2_) where the self-heating rate passes above 15 °C min^−1^. The peak temperature during thermal runaway is labelled as T_3_. In the temperature profile for all cells, a small exothermic reaction was consistently recorded around 95 °C that eventually reduced to below the detection limit. As this exotherm was consistent across every cell tested, it is likely to be an inactive material in the cell. This may be attributed to the separator: a low molecular weight polyethylene separator could conceivably melt at 95 °C. Melting, or even shrinking, of the separator brings the electrodes into contact to cause a heat-producing short.^36^ However, the temperature is likely too low, as polypropylene is a more commonly used separator, and melts at 130–140 °C.^37^ Instead, adhesive or inner casing in the cell casing breaking down, releasing hot gasses from the cell that register as an exothermic reaction is suggested as a likely cause. The recommended heater bar setting for sealing the pouch cells used in this work is 160 °C for 3 seconds. It is expected that the melting point of the adhesive is below this temperature. Referring to both the slow heating conditions and build-up of pressure inside the cell, it is very possible that 95 °C is the breaking point for the pouch cell seal. As summarised in Fig. 3A, the polycrystalline material shows a self-heating onset approximately 15 °C higher than the single-crystal material. This could be due to a difference in specific surface area between the two materials. The single-crystal material is made of primary particles with a diameter of 1–2 μm, whereas the polycrystalline material has a wider range of particle sizes, and a *D*_50_ of 9.6 μm (Fig. 7). Cathode particle morphology has been shown to influence the safety behaviour of cells and is the main difference between the two cells studied here.^20,21^

Shown in Fig. 3A, T_3_ during thermal runaway was approximately 30 °C higher for the pristine polycrystalline materials than the pristine single-crystal cell. One of the later stages of thermal runaway is decomposition of the cathode and reactions with the electrolyte. It has been postulated that as the temperature of the cell accelerates, the particles start to fracture along the grain boundaries of the secondary agglomerates.^23^ The fresh surface of the interior of the particles is exposed to the electrolyte and reactive gases. The heat and gas produced from the reactions between the particle–electrolyte interface drives further fracturing, exposing more surface area and allowing more exothermic reactions to take place. This would increase the temperature and continue the domino effect of thermal runaway.

After cycling and characterisation of electrochemical behaviour of both cell types, the same calorimetry testing was performed using cycled cells to compare changes in the characteristic failure temperatures with their pristine counterparts. As with the pristine cells, an exothermic reaction was consistently observed at 95 °C. For the polycrystalline cells, T_1_ reduced by approximately 10 °C, from 151 °C to 141 °C after cycling (Fig. 3A). This indicates a reduction of the thermal stability due to cycle ageing. This effect may be the result of cycling-induced particle fracture, wherein the greater specific surface area of the cathode could give more facile kinetics for reactions with the electrolyte. A reduction in T_3_ was also observed and was consistent across both types of material. Although there is high variability across cells, and the peak temperatures are broadly within error of each other, some trends can still be observed. The difference between the pristine and cycled cells may be the result of a reduction in energy available in the cell due to capacity loss with cycling. This loss in capacity reflects a loss in reactivity of the component chemicals, causing a reduction in the peak thermal runaway temperature. This largely fits the trend observed in previous works for cells cycled at ambient temperature within their specified voltage range.^38,39^

The polycrystalline cells also gave a decrease in the thermal runaway initiation point, which is defined in this work as the temperature where d*T*/d*t* exceeds 15 °C min^−1^. Like the onset of self-heating temperature, a reduction in the thermal runaway initiation point means a loss of thermal stability in the components.

For the single-crystal cells, the opposite trend was observed in terms of thermal stability, with an increase in both the onset of self-heating and the initiation of thermal runaway following cycle ageing (Fig. 3C and E). The exact reason for this is unclear, but this phenomenon should be considered alongside the lack of morphological changes that occur at the particle level during cycling. With no particle cracking and fragmentation, the cathode specific surface area should remain relatively constant before and after cycling. At the same time there was a reduction in the available energy in the cell, degradation of some of the components to less reactive products, and formation of a more stable SEI layer. These effects may have led to an increase in thermal runaway initiation temperatures. The single-crystal cells performed more cycles than the polycrystalline cells. As a result, there might have been a relatively large degree of anode-side degradation, which could have led to a thicker and more stable SEI layer. This idea contradicts the work of Börner *et al.*, who showed that a thicker SEI layer is likely to contain more metallic lithium, which in turn reduces the onset temperature of self-heating.^38^ However, in this work, the accelerated degradation of the polycrystalline cells may have contributed to negative electrode side degradation *via* cross talk mechanisms. For example, greater internal resistance caused by particle fracture in the polycrystalline cells could lead to increased anode slippage and more metallic lithium in the SEI. Whereas the SEI in the single-crystal cells may be composed primarily of less reactive degradation products. Also, it's important to note that the differences between onset temperature before and after cycling for both sets of morphology are quite small, and there is some variability in the onset temperature between repeat samples, as indicated in the error bars in Fig. 3A.

While the cells investigated in this work have been fabricated for research purposes, it is important to consider the findings here in the wider context of how the characteristic temperatures might map onto the ability of the investigated cells to pass current energy storage regulations. T_1_, the self-heating onset temperature, is used to evaluate the thermal stability of the cell under heating abuse conditions. A high enough T_1_ temperature is necessary to pass the thermal ramp and fire exposure tests present in testing standards such as UL2580, IEC 62660-2 and SAE J2464.^40–42^

T_2_, the initiation of thermal runaway, is typically viewed as the “point of no return”, where a catastrophic failure is almost impossible to stop. In an industrial battery safety assessment setting, T_2_ can used to calculate the time margin from when a cell fault is detected to when a thermal runaway event is going to happen. A higher T_2_ temperature not only means a greater safety margin when a cell is self-heating but also increases resistance to propagation. Like T_1_, a sufficient T_2_ temperature is needed to pass thermal abuse regulation present in UL2580, IEC 62660-2 and SAE J2464.^40–42^

T_3_, the maximum temperature reached during thermal runaway is often used to describe the severity of a thermal runaway event. A higher T_3_ temperature means greater solid-body energy release from a cell undergoing thermal runaway, while the ejecta is also likely to be a higher temperature, where the ejecta could do on to damage and melt other parts of a battery pack, device or vehicle. A higher T_3_ also means any gas released will be at a higher pressure, which has the knock-on effect of influencing vent and battery enclosure design. Understanding T_3_ is vital for designing against propagation, a key requirement in the Chinese standard GB 38021-2025 and the European aviation standard EASA MOC-5 SC VTOL.^43,44^

### Post-cycling CT

**Fig. 4:**
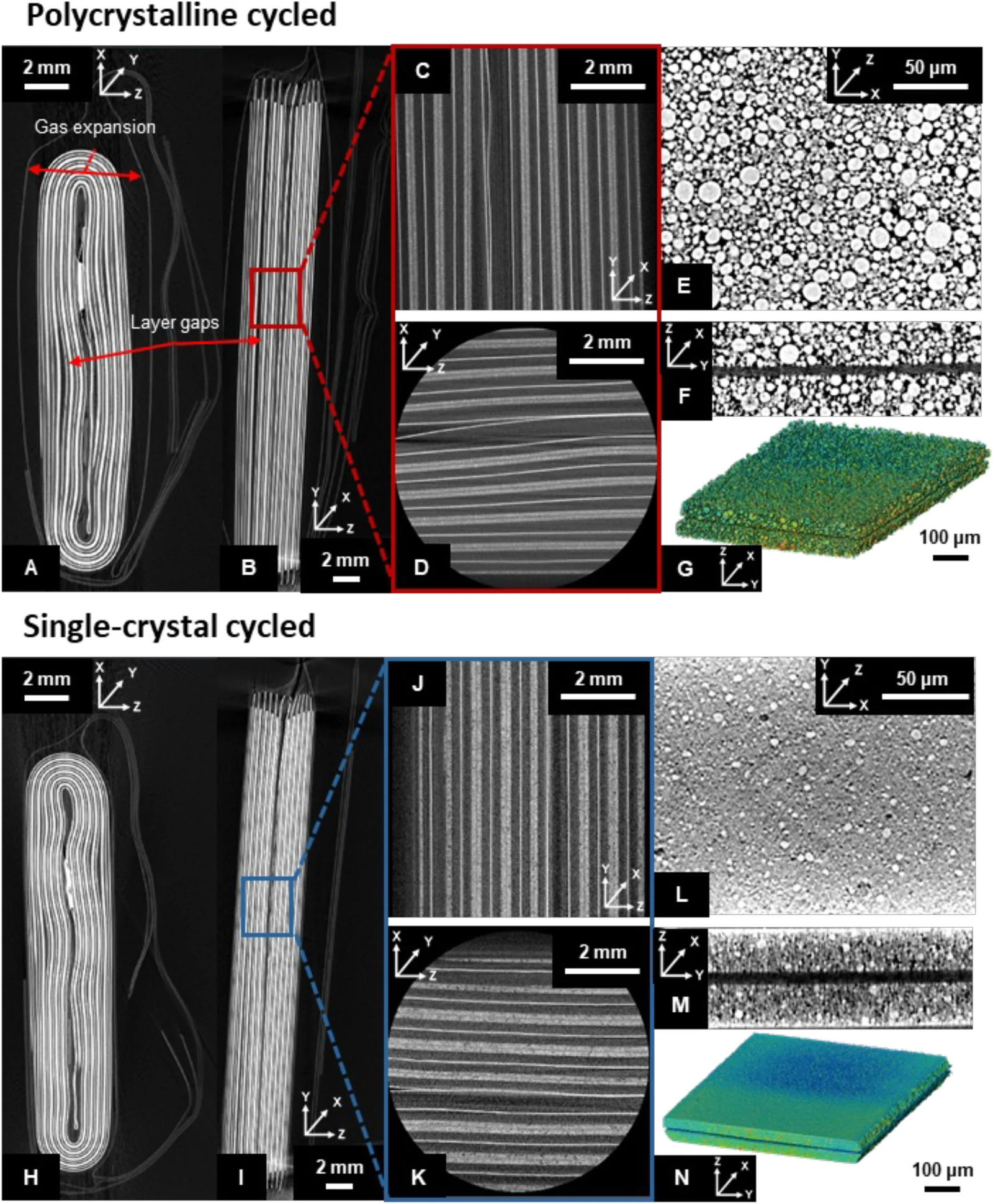
X-ray CT images taken from polycrystalline and single-crystal cells after cycling to 80% capacity retention. (A and B) Orthoslices taken from a representative polycrystalline cell post-cycling. (C and D) Orthoslices taken from a 4× ROI scan of a polycrystalline cell post-cycling. (E and F) Orthoslices taken from a 40× *ex situ* sample from a polycrystalline cell post-cycling, (G) shows a volume rendering of the same sample. (H and I) Orthoslices taken from a representative single-crystal cell post-cycling. (J and K) Orthoslices taken from a 4× ROI scan of a single-crystal cell post-cycling. (L and M) Orthoslices taken from a 40× *ex situ* sample from a single-crystal cell post-cycling, (N) shows a volume rendering of the same sample.

Multiscale CT imaging was performed on all cells in this work. Full cell scans were carried out to image the larger-scale effects of cycling and thermal runaway. For more detailed investigation, higher resolution region-of-interest (ROI) scans were performed *in situ* (without opening) near the centre of each cell. Fig. 4A and B show the virtual slices taken both along the horizontal and vertical planes of the respective cells, revealing the damage to the jellyroll structure during thermal failure. Full cell CT of the pristine cells can be found in S1.

**Fig. 5:**
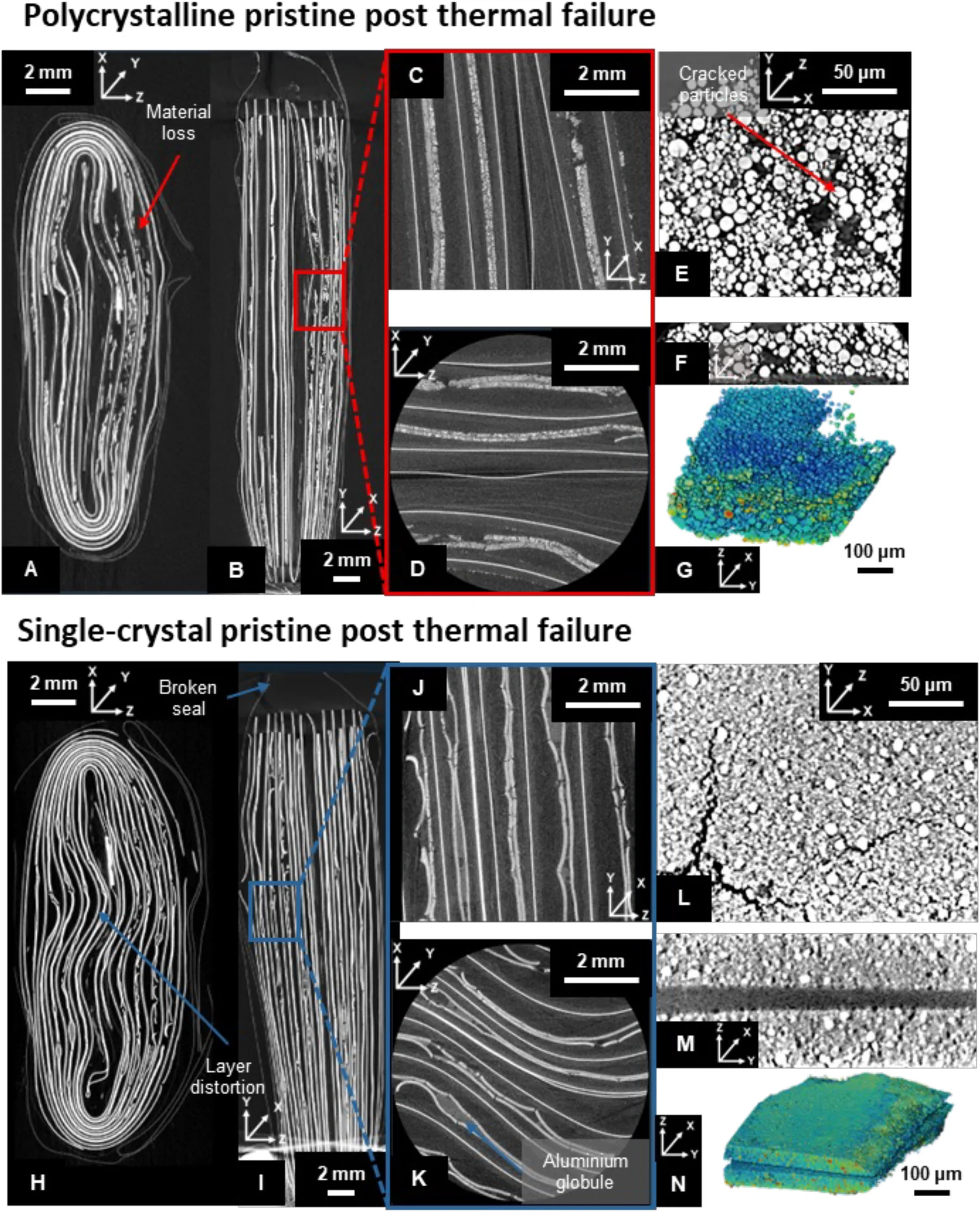
X-ray CT images taken from uncycled polycrystalline and single-crystal cells post-thermal runaway. (A and B) Orthoslices taken from a representative polycrystalline cell post-failure. (C and D) Orthoslices taken from a 4× ROI scan of a polycrystalline cell post-failure. (E and F) Orthoslices taken from a 40× *ex situ* sample from a polycrystalline cell post-failure, (G) shows a volume rendering of the same sample. (H and I) Orthoslices taken from a representative single-crystal cell post-failure. (J and K) Orthoslices taken from a 4× ROI scan of a single-crystal cell post-failure. (L and M) Orthoslices taken from a 40× *ex situ* sample from a single-crystal cell post-failure, (N) shows a volume rendering of the same sample.

The CT inspection showed more gas expansion in the polycrystalline cells than the single-crystal. The representative polycrystalline cell has expanded from 3.7 mm to 6.0 mm, an increase of 62%. The representative single-crystal cell only swelled by 0.2 mm, from 3.5 mm to 3.7 mm, an increase of just 6%. The CT scans in Fig. 5A and H also showed greater loss of contact after cycling in the polycrystalline cell, exacerbated by gas evolution, between the layers along the long side of the cell. Although the single-crystal cell in Fig. 4A showed some defects and gaps between the layers, the process of repeated charge–discharge cycling appears to have had less of an effect on the contact between layers and capacity retention.

Little change was observed from the micro-CT in the single-crystal material after cycling. This could simply have been due to the insufficient resolution of the instrument, making it difficult to probe at the particle level. For the polycrystalline cathode material, a greater degree of particle fracturing and cracking was observed after cycling compared to the pristine material, which is shown quantitatively in the particle size distribution in Fig. 7. This matches with observations made in previous work that particle cracking is a key form of material degradation in Li-ion batteries.^45,46^

### X-ray CT comparison of pristine single-crystal and polycrystalline cells after failure

After thermal failure, both polycrystalline and single-crystal cells were CT scanned at various length scales. Fig. 5 shows the full-cell, lower resolution virtual-slices and 3D renderings for cells with both particle morphologies after cycling. Multiscale CT imaging was performed on all cells in this work. Full cell scans were carried out to image the larger-scale effects of cycling and thermal runaway. For more detailed investigation, higher resolution region-of-interest (ROI) scans were performed *in situ* (without opening) near the centre of each cell. Fig. 5A and B show the virtual slices taken both along the horizontal and vertical planes of the respective cells, revealing the damage to the jellyroll structure during thermal failure. Full cell CT of the pristine cells can be found in S1.

All the failed cells showed significant changes to the internal architecture, compared with the pristine cells, as would be expected. However, the nature of the location of the changes is interesting to note; it was observed that, across all types of the cell, the corners of the jellyroll force the gases through the long sides of the cell, as well as up towards the tab seals (Fig. 5A–B and H–I). This is clear from the fracturing of the electrode layers along the long sides of the cells, whereas the corners have stayed relatively intact. Areas where the electrode layers have deformed and separated from each other, could be funnelling gasses towards the tab seal, a weak point in the cell packaging. There is also evidence across both types of cells of the cathode layer delaminating and fracturing.

Qualitatively, the polycrystalline cells show a greater degree of damage throughout. There are several areas where the cathode has delaminated away from the aluminium current collector. Both cell types show evidence of aluminium melting, with several globules present caught in between the cathode layers of the polycrystalline cell. Aluminium melts at 660 °C; therefore, the centre of the cell, or the gasses produced, must have reached or exceeded this temperature. There are also some globules of aluminium closer to the edges of the single-crystal cell, but not near the centre. As there is an overall lack of material near the centre of the single-crystal cell, it is likely that the produced gasses, with most of the heat and kinetic forces, of the thermal runaway event has vented much of this material out of the top of the cell, where the tab seal was seen to have failed (Fig. 5I). Due to the thermocouples being located on the exterior of the cell, the internal temperature changes are inferred from the differences visible after failure in the full cell CT scans. The distinct differences between the two cathode morphologies suggest different internal temperatures and distributions of material and heat during failure.

The failed cells were then dismantled, and a sample was collected from the cathode from the centre of each. A tab geometry was used for sample preparation to allow for ease of fabrication and to reduce the risk of laser damage.^47^ Micro-CT was performed on cathode samples from both types of cell to produce higher resolution datasets investigate changes at the microstructural level during failure. Fig. 5E, F and L, M shows the orthoslices of *XY* and *XZ* orientations. Even at pixel sizes of approximately 200 nm, it was difficult to clearly distinguish the discrete particles of the single-crystal materials, whereas the secondary agglomerates in the polycrystalline NMC are clearly visible. However, we can still observe some larger scale features, such as cracks that propagate throughout the single-crystal NMC coating. For the polycrystalline cell, there are visually more cracked and damaged particles, particularly for the larger agglomerates. There also appear to be voids between the clusters of remaining particles, possibly due to outgassing from the cathode side. However, this could just be a product of damage sustained during cell dismantling and sample preparation. Overall, it was observed that the polycrystalline particles have broken apart during failure and the surface area has increased. This observation adds weight to the argument that the difference in peak temperature during failure between the two material morphologies is due to particle fracture during failure at high temperatures.

### Post-cycling ARC X-ray CT comparison

**Fig. 6:**
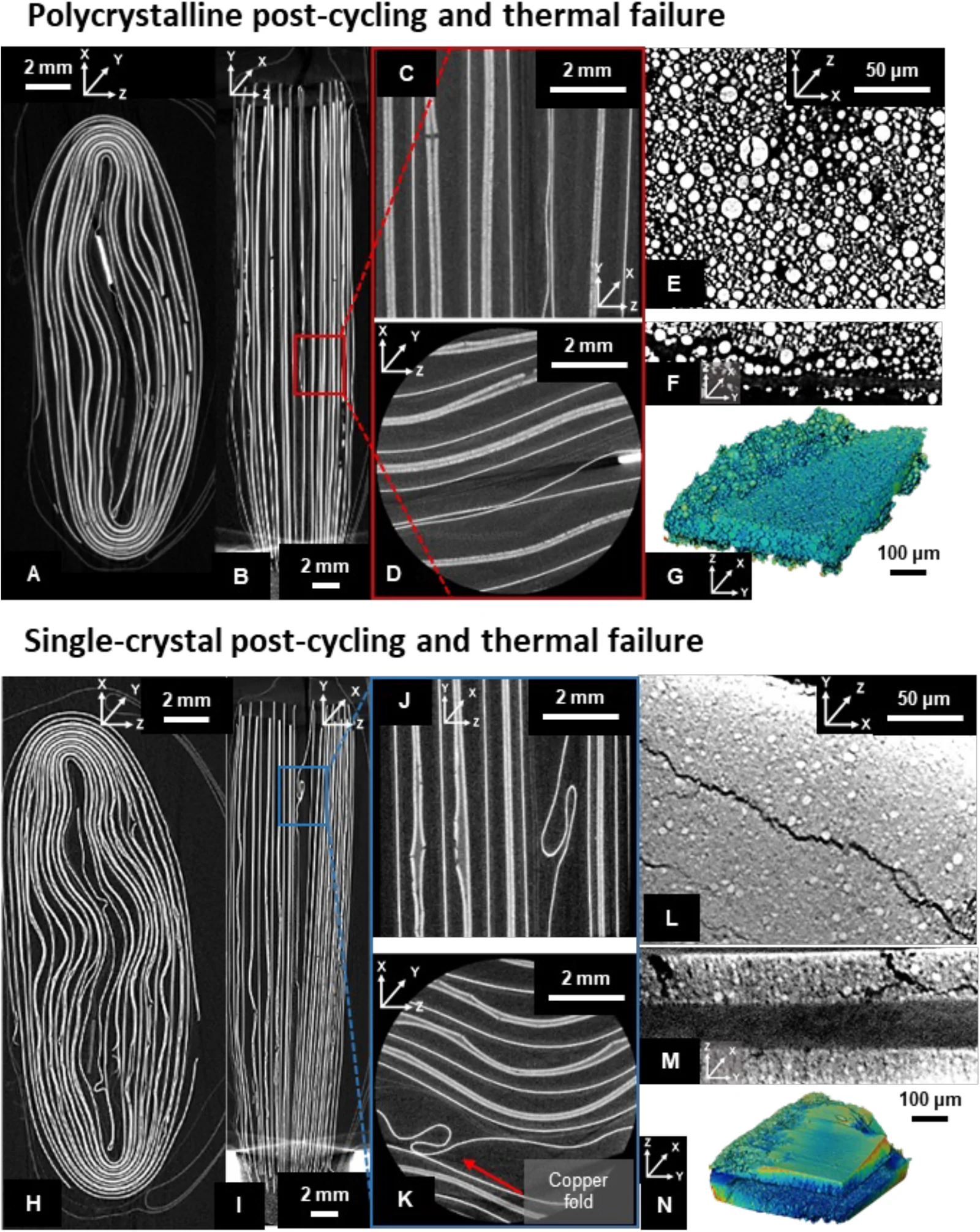
X-ray CT images taken from polycrystalline and single-crystal cells after cycling to 80% capacity retention. (A and B) Orthoslices taken from a representative polycrystalline cell post-cycling and failure. (C and D) Orthoslices taken from a 4× ROI scan of a polycrystalline cell post-cycling and failure. (E and F) Orthoslices taken from a 40× *ex situ* sample from a polycrystalline cell post-cycling and failure, (G) shows a volume rendering of the same sample. (H and I) Orthoslices taken from a representative single-crystal cell post-cycling and failure. (J and K) Orthoslices taken from a 4× ROI scan of a single-crystal cell post-cycling and failure. (L and M) Orthoslices taken from a 40× *ex situ* sample from a single-crystal cell post-cycling and failure, (N) shows a volume rendering of the same sample.

After cycled cells underwent an ARC safety assessment, the same multiscale X-ray CT analysis was performed on the failed cells and the recovered cathode material. Fig. 6 shows virtual-slices and volume renderings from two representative cells, one of each cathode morphology.

The whole-cell CT for both single- and polycrystalline cells showed a lower degree of damage overall compared to the pristine failed cells, with less delamination and fracturing of the electrode layers. Notably, in both polycrystalline and single-crystal aged cells there were some areas where the aluminium had melted and distorted the anode electrodes, suggesting the internal temperature of the cell reached around 660 °C. This contrasts with the pristine cells, which showed extensive aluminium melting and reforming. In the polycrystalline cell, there was some distortion along the long sides of the cell where there was evidence of electrode displacement and gaps opening due to cycling. This was even more evident on the single-crystal cell, where regions near the centre of the cell where gases have opened gaps during cycling also showed the greatest degree of electrode delamination and displacement of the layers. The post-cycling CT suggests that gaps that had grown between the electrode layers due to gas formation with cycling, acted as channels for the gasses formed during failure, focussing the force of cell failure in these areas.

The *ex situ* high magnification CT, showing the particle structure, revealed significant damage to both morphologies after failure. From the polycrystalline cathode, there was visible cracking, especially around the larger particles. There was also a greater degree of porosity observed, with more space between the particles and less material between. The greater degree of damage seen at the particle scale contrasts with the macro level, suggesting the particles cracked during the cycling process. The single-crystal material, similar to the 40× CT scans taken after the pristine ARC tests (Fig. 5L), showed larger scale cracks across the sample, both across the face of the sample but also through the *z*-plane, but the discrete crystals remain largely intact.

**Fig. 7:**
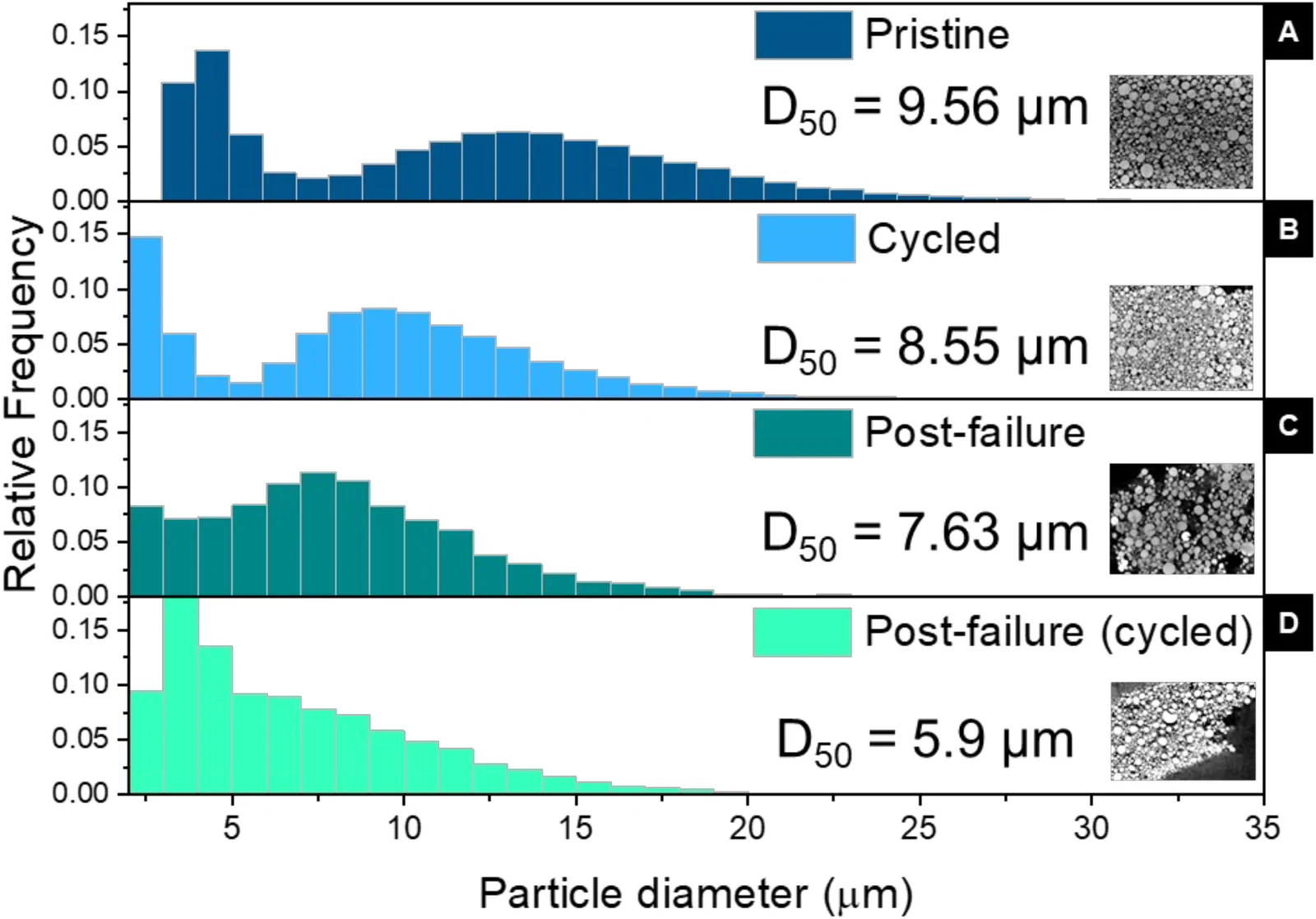
Particle size distribution of polycrystalline materials. (A) Pristine, (B) cycled to 80% capacity retention, (C) uncycled material post-thermal runaway, (D) cycled material post-thermal runaway.

Although the resolution is not sufficient to apply common image-based analysis techniques to the single-crystal materials, an analysis of the particle size distribution was applied to the polycrystalline materials as-received, after failure, after cycling and after cycling and failure (Fig. 7).

The particle size distributions show that the pristine and cycled polycrystalline cathode have a bimodal distribution. The pristine has major peaks around 3–4 μm and a broader peak around 13 μm, whereas the cycled material has peaks at lower particle diameters, >3 μm and 9–10 μm respectively. The difference in *D*_50_ (median) particle diameter between the pristine and cycled cathodes suggest some cracking has occurred during cycling. We observe that both the smaller and larger particles reduced in particle size, even though the larger particles are likely to undergo more strain during expansion and shrinkage with cycling.^48^ The failed materials show a broadly monomial distribution, where the main peak from the pristine post-failure material was around 8 μm, and the cycled post-failure material giving a major peak in the 3–4 μm region. In all cases, the thermal runaway process led to a fracturing of the polycrystalline particles, as has been observed in other works.^20,49^ It was also observed that the failed materials showed a singular peak, with a loss of the much of the smaller particles (<3 μm). This could be due to combined heat and force of the cell failure leading to an ejection of the particles, which formed some of the dust that was observed in the calorimeter chamber after the failure tests. Finally, it is also important to note that only material that has largely remained intact is usable for *ex situ* CT imaging after thermal runaway. This introduces a degree of bias, where the particles that can be mounted for CT inspection are likely to be less damaged than particles that have lost adhesion that have been removed from the current collector. Therefore, it is likely that the ‘true’ particle size distribution of the material after failure is somewhat less than the results presented here. However, that does not compromise the trends observed from the CT data in this work.

**Fig. 8:**
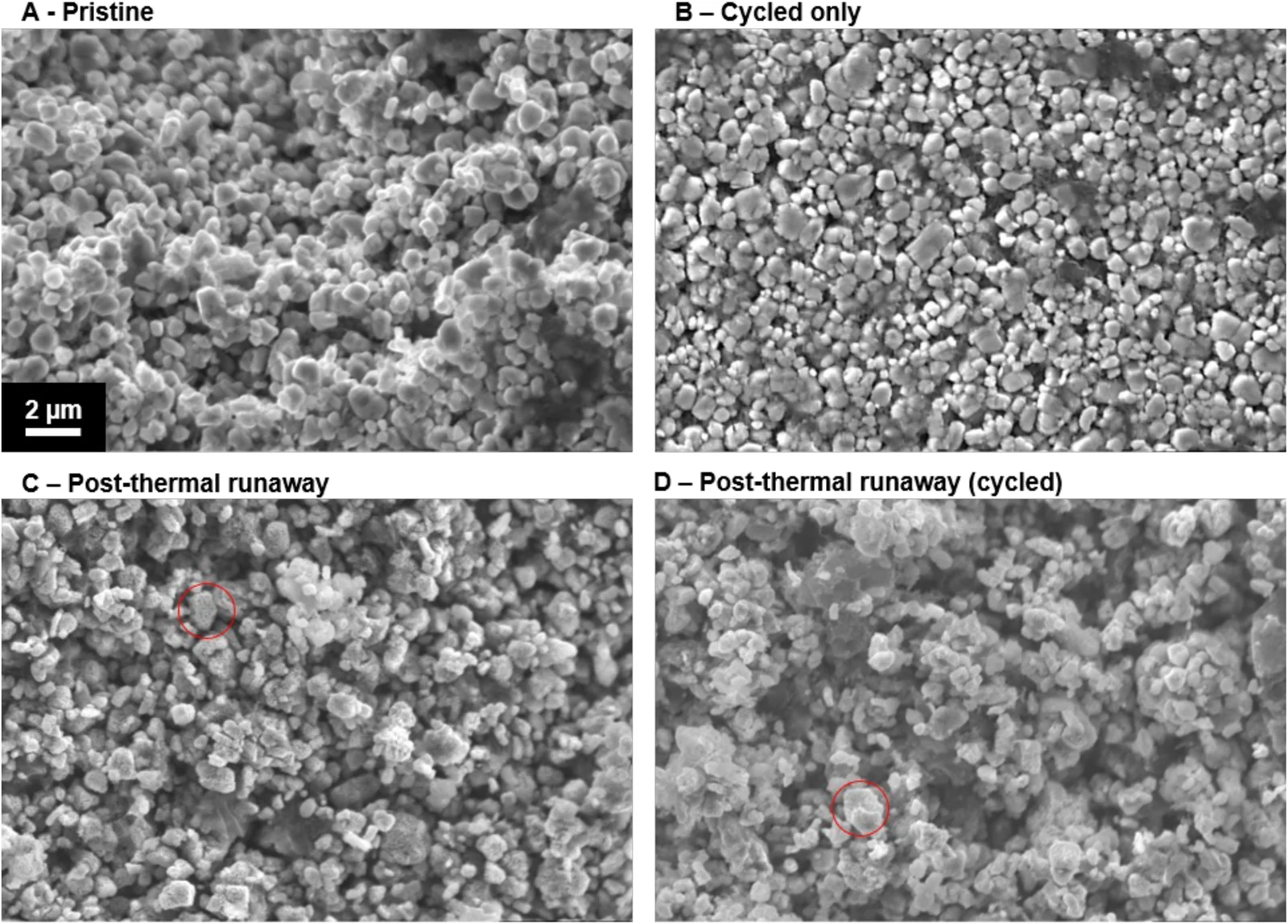
SEM images taken at approximately 5k× magnification in different conditions. (A) Pristine, (B) cycled to 80% capacity retention, (C) uncycled material post-thermal runaway, (D) cycled material post-thermal runaway. The red circles in (C) and (D) highlight particle where there is some intraparticle porosity visible. Images have been edited for brightness and contrast to improve clarity.

While the CT instruments available for this work cannot sufficiently resolve the particle sizes in the single-crystal materials, scanning electron microscopy (SEM) can be used to qualitatively evaluate the particle condition of single-crystal cathodes in different conditions (Fig. 8).

The images in Fig. 8 provide a surface-level perspective that can be used to evaluate the damage that the particles have undertaken during cycling and thermal runaway. Images taken of the same materials at the lower magnification can be found in Fig. S1. The pristine and cycled materials (Fig. 8A and B) shows broadly an arrangement of similar-sized smaller particles, approximately 1 µm diameter, with some larger particles, approximately 2 µm diameter, dispersed between them. This observation is expected, as the key function of single-crystal materials is that they do not fracture due to cycle ageing. The post-thermal runaway materials show a similar particle size and shape to the electrode that have not undergone thermal runaway, however there is some evidence of changes in the particles. As highlighted in red in Fig. 8C and D, there are some larger particles which appear to be agglomerates of even smaller primary particles. From the SEM images, the thermal runaway process has led to some particle damage and swelling of the structure, although on a much more subtle level compared to the polycrystalline particles. It is unclear whether this slight fracturing of the particle occurred during leading up to the thermal runaway, and in any way played a role in the reactions driving the self-heating process, or occurred due high temperatures and gas release in the final catastrophic failure of thermal runaway. The gaps created within the particle appear to be of the order of nanometres in diameter, if the particles did fracture slightly to reveal these channels during the self-heating process, it is likely that they play a minor role in the reactivity of the cathode relative to the fracturing seen in the polycrystalline materials.

## Conclusions

We have used multiscale post-mortem imaging both after cycling and thermal failure, to provide insight into the effect of cathode active material crystal morphology and microstructure on battery safety over the course of the cell lifetime. The electrochemical differences between polycrystalline and single-crystal cathode materials were explored by assessing the degradation of both cathode morphologies. It was found that the single-crystal material showed superior cycle life to the polycrystalline material, taking approximately 50% more cycles to reach 80% capacity fade. The polycrystalline cell gave a two-phase capacity-fade profile, where the capacity fades at a constant rate for the first 445 cycles, before accelerating following a ‘knee-point’. The single-crystal cell showed a steady rate of degradation throughout its 670 cycle lifetime. Visual evaluation and macro X-ray CT showed that the polycrystalline cell produced far more gas with cycling than the single-crystal cell. Post-mortem micro-CT showed that there was evidence of particle cracking in the polycrystalline cell, however the resolution was not sufficient to make out the specifics of the single-crystal particle structure.

From a battery safety perspective, the polycrystalline cell showed a greater self-heating temperature than the single-crystal cell in the pristine state, but also a higher peak temperature during thermal runaway. The results suggest that pristine polycrystalline cathode materials have a greater thermal stability (prior to thermal runaway) than single-crystal alternatives of the same chemistry, but will also have a more energetic failure, increasing the risk of propagation in a battery pack. After cycling, the polycrystalline cells showed a reduction in thermal stability, but also a reduction in peak temperature. The reasons for this are unclear, as a reduction in thermal stability of aged cells is often attributed to Li-plating on the anode. This is still a possibility here, as some small, localised areas of Li plating were observed in the dismantled polycrystalline cells after cycling. Cross talk between the faster degrading polycrystalline cathode and accompanying anode may have initiated plating and affected the safety characteristics. On the other hand, the high magnification CT showed that the cycle ageing of the polycrystalline cells had also led to a reduction in the mean particle size distribution, suggested that cracking occurred during cycling. The reason for the reduction in the self-heating temperature of aged cells could be due to the greater surface area of the cracked cathode materials. The increased surface area then leads to more facile kinetics between cathode and electrolyte reactions. The single-crystal cells showed a smaller reduction in the peak temperature with ageing and, in contrast to the polycrystalline cathode, an increase in the self-heating temperature. Although the reasons for this were not clear, the hypothesis is presented that this observation is due to a lack of particle cracking and growth of a more stable SEI layer. Where both effects lead to a greater overall thermal stability in the single-crystal cells. This theory is strengthened by SEM imaging, which shows no obvious particle cracking in the single-crystal materials. However, further work using higher resolution imaging techniques as well as failure studies on single-crystal materials that have been aged under a greater variety of ageing environments, and thus inducing different degradation modes, are necessary to be certain.

Overall, this work suggests that single-crystal materials offer a marginal improvement in safety characteristics for end-of-life battery cells. This also highlights the need to understanding the links between ageing and safety if cells are to be used in second-life applications, as these results suggest particle cracking may reduce the thermal stability of positive electrode materials with use. CT imaging can play a role in determining the state-of-safety of cells, as a tool for identifying macro-scale differences at the end of life that play in role in safety performance.

## Conflicts of interest

There are no conflicts to declare.

## Supplementary Material

EB-OLF-D6EB00099A-s001

## Data Availability

The datasets generated during and/or analysed during the current study are not publicly available but are available from the authors on reasonable request. Supplementary information (SI) containing all the SEM images taken is available. See DOI: https://doi.org/10.1039/d6eb00099a.

## References

[cit1] Hasan M. M., Haque R., Jahirul M. I., Rasul M. G., Fattah I. M. R., Hassan N. M. S., Mofijur M. (2025). J. Energy Storage.

[cit2] Kim T., Song W., Son D.-Y., Ono L. K., Qi Y. (2019). J. Mater. Chem. A.

[cit3] Zhou L., Zhang K., Hu Z., Tao Z., Mai L., Kang Y. M., Chou S. L., Chen J. (2018). Adv. Energy Mater..

[cit4] Etacheri V., Marom R., Elazari R., Salitra G., Aurbach D. (2011). Energy Environ. Sci..

[cit5] Ould Ely T., Kamzabek D., Chakraborty D. (2019). Front. Energy Res..

[cit6] Preger Y., Torres-Castro L., Rauhala T., Jeevarajan J. (2022). J. Electrochem. Soc..

[cit7] Carter R., Klein E. J., Atkinson R. W., Love C. T. (2019). J. Power Sources.

[cit8] Waldmann T., Wohlfahrt-Mehrens M. (2017). Electrochim. Acta.

[cit9] Ren D., Hsu H., Li R., Feng X., Guo D., Han X., Lu L., He X., Gao S., Hou J., Li Y., Wang Y., Ouyang M. (2019). eTransportation.

[cit10] Kirchner-Burles C., Fordham A., Reid H. T., Johnson M., Buckwell M., Iacoviello F., Coke K., Jervis R., Hinds G., Shearing P. R., Robinson J. B. (2025). J. Power Sources.

[cit11] Liu J., Duan Q., Ma M., Zhao C., Sun J., Wang Q. (2020). J. Power Sources.

[cit12] Wang Z., Zhao Q., Wang S., Song Y., Shi B., He J. (2024). J. Power Sources.

[cit13] Qian G., Zhang Y., Li L., Zhang R., Xu J., Cheng Z., Xie S., Wang H., Rao Q., He Y., Shen Y., Chen L., Tang M., Ma Z. F. (2020). Energy Storage Mater..

[cit14] Langdon J., Manthiram A. (2021). Energy Storage Mater..

[cit15] Lüther M. J., Jiang S. K., Lange M. A., Buchmann J., Gómez Martín A., Schmuch R., Placke T., Hwang B. J., Winter M., Kasnatscheew J. (2024). Small Struct..

[cit16] Jangde A., Kumar M., Tuğrul Gülenç İ., Wheatcroft L., Inkson B. J. (2025). Batteries Supercaps.

[cit17] Duan J., Wu C., Cao Y., Huang D., Du K., Peng Z., Hu G. (2017). J. Alloys Compd..

[cit18] Wang L., Wu B., Mu D., Liu X., Peng Y., Xu H., Liu Q., Gai L., Wu F. (2016). J. Alloys Compd..

[cit19] Liu Y., Dong J., Guan Y., Wang X., Chen Z., Gao M., Guo S., Yan K., Lu Y., Wang M., Li N., Su Y., Wu F., Chen L. (2025). Adv. Mater..

[cit20] Finegan D. P., Scheel M., Robinson J. B., Tjaden B., Michiel M. Di, Hinds G., Brett D. J. L., Shearing P. R. (2016). Phys. Chem. Chem. Phys..

[cit21] Jiang J., Dahn J. R. (2004). Electrochim. Acta.

[cit22] Geder J., Hoster H. E., Jossen A., Garche J., Yu D. Y. W. (2014). J. Power Sources.

[cit23] Chiba K., Yoshizawa A., Isogai Y. (2020). J. Energy Storage.

[cit24] Song Y., Cui Y., Li B., Geng L., Yan J., Zhu D., Zhou P., Zhou J., Yan Z., Xue Q., Tang Y., Xing W. (2023). Nano Energy.

[cit25] Kong X., Zhang Y., Li J., Yang H., Dai P., Zeng J., Zhao J. (2022). Chem. Eng. J..

[cit26] Wood V. (2018). Nat. Rev. Mater..

[cit27] Patel D., Reid H., Ball S., Brett D. J. L., Shearing P. R. (2023). Johnson Matthey Technol. Rev..

[cit28] Wu Y., Saxena S., Xing Y., Wang Y., Li C., Yung W. K. C., Pecht M. (2018). Energies.

[cit29] Patel D., Reid H., Ball S., Brett D. J. L., Shearing P. R. (2023). Johnson Matthey Technol. Rev..

[cit30] Heenan T. M. M., Llewellyn A. V., Leach A. S., Kok M. D. R., Tan C., Jervis R., Brett D. J. L., Shearing P. R. (2020). Adv. Sci..

[cit31] Attia P. M., Bills A., Brosa Planella F., Dechent P., dos Reis G., Dubarry M., Gasper P., Gilchrist R., Greenbank S., Howey D., Liu O., Khoo E., Preger Y., Soni A., Sripad S., Stefanopoulou A. G., Sulzer V. (2022). J. Electrochem. Soc..

[cit32] Zhang H., Dong J., Lu Y., Liu Y., Hao J., Li N., Chen G., Huang Q., Su Y., Wu F., Chen L. (2025). Nano Energy.

[cit33] Dose W. M., Xu C., Grey C. P., De Volder M. F. L. (2020). Cell Rep. Phys. Sci..

[cit34] Yang X. G., Leng Y., Zhang G., Ge S., Wang C. Y. (2017). J. Power Sources.

[cit35] Harris S. J., Lu P. (2013). J. Phys. Chem. C.

[cit36] Sierra J. D., Osswald T. A. (2000). J. Plast. Film Sheeting.

[cit37] Wu Y., Wu Q., Sun M., Zeng Z., Cheng S., Xie J. (2025). Adv. Funct. Mater..

[cit38] Börner M., Friesen A., Grützke M., Stenzel Y. P., Brunklaus G., Haetge J., Nowak S., Schappacher F. M., Winter M. (2017). J. Power Sources.

[cit39] Xie S., Ren L., Yang X., Wang H., Sun Q., Chen X., He Y. (2020). J. Power Sources.

[cit40] UL Solutions , UL2580 Batteries for Use in Electric Vehicles, Underwriters Laboratories (UL), 3rd edn, 2020

[cit41] IEC , IEC 62660-3:2022 Secondary lithium-ion cells for the propulsion of electric road vehicles - Part 3: Safety requirements, International Electrotechnical Commission, 3rd edn, 2022

[cit42] SAE International , Electric and Hybrid Electric Vehicle Rechargeable Energy Storage System (RESS) Safety and Abuse Testing, SAE International, 400 Commonwealth Drive, Warrendale, PA, United States, 2021

[cit43] Standardization Administration of China , GB 38031-2025 Electric vehicles traction battery safety requirements, Standardization Administration of China, 2025

[cit44] EASA , MOC-5 SC VTOL: Fifth Publication of Proposed Means of Compliance with the Special Condition VTOL, European Union Aviation Safety Agency, 5th edn, 2025

[cit45] Liu W., Oh P., Liu X., Lee M. J., Cho W., Chae S., Kim Y., Cho J. (2015). Angew. Chem., Int. Ed..

[cit46] Yan P., Zheng J., Liu J., Wang B., Cheng X., Zhang Y., Sun X., Wang C., Zhang J. G. (2018). Nat. Energy.

[cit47] Tan C., Daemi S., Heenan T., Iacoviello F., Leach A. S., Rasha L., Jervis R., Brett D. J. L., Shearing P. R. (2020). J. Electrochem. Soc..

[cit48] Parks H. C. W., Boyce A. M., Wade A., Heenan T. M. M., Tan C., Martínez-Pañeda E., Shearing P. R., Brett D. J. L., Jervis R. (2023). J. Mater. Chem. A.

[cit49] Patel D. , Robinson J. B., Ball S. and Brett D. J. L.. 10.1149/1945-7111/ab7fb6

