# Supplementary material for: A multiscale X-ray CT study of degradation and safety behaviour of different Li-ion battery positive electrode morphologies
Source: EES Batter. 2026 Sep 15. Online ahead of print. doi: 10.1039/d6eb00099a (PMC13576674; doi:10.1039/d6eb00099a)
Supplement: Supplementary file 1 [file EB-OLF-D6EB00099A-s001.pdf]

Supplementary Information (SI) for EES Batteries.
This journal is © The Royal Society of Chemistry 2026

SUPPLEMENTAL INFORMATION

A multiscale X-ray CT study of degradation and safety behaviour of different Li-ion battery positive electrode morphologies

Hamish T. Reid, Drasti Patel, Will J. Dawson, James B. Robinson, Rhodri Jervis, Paul R. Shearing

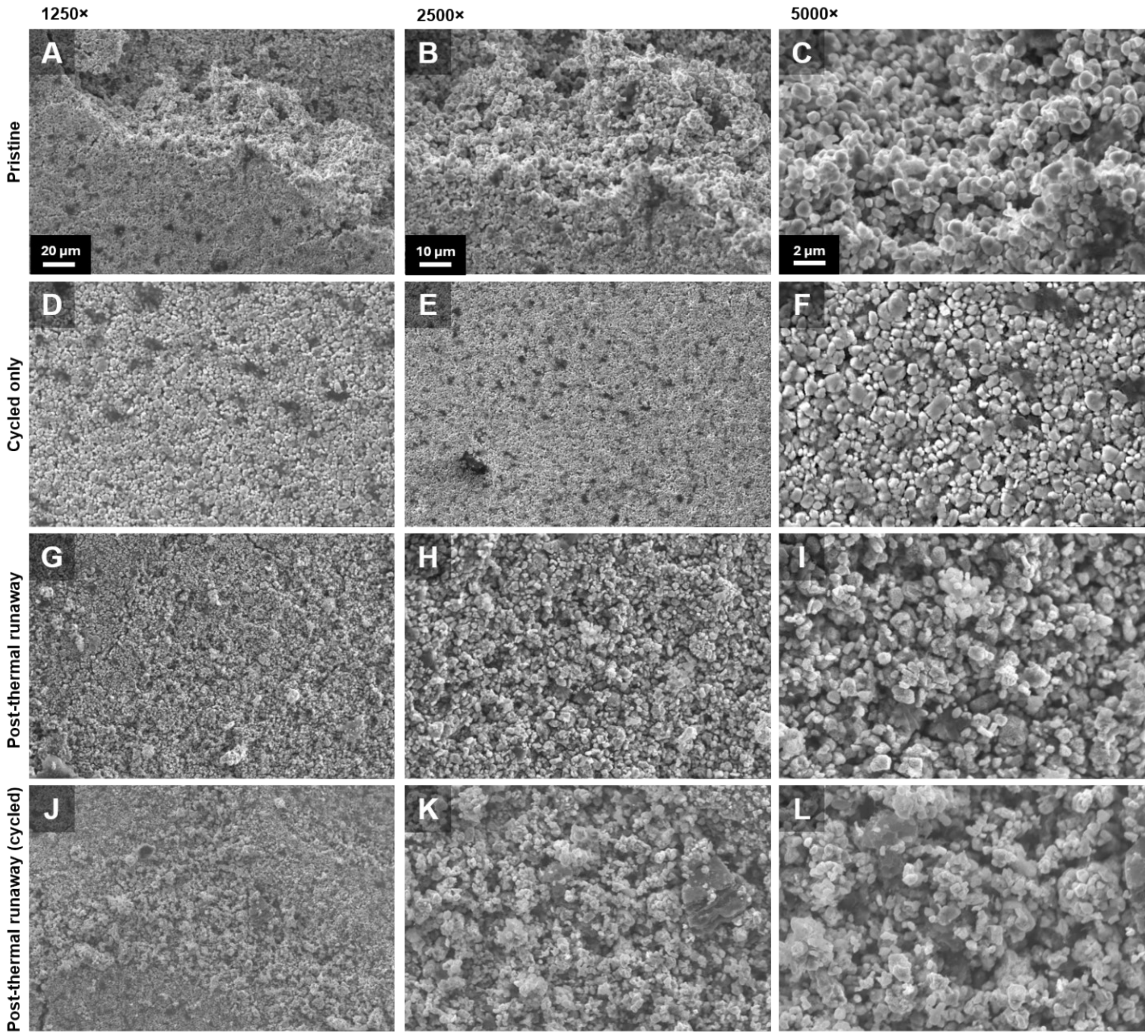

Figure S1 SEM images taken of single-crystal cathode material harvested from cells in different conditions. A-C images of pristine material taken at 1250×, 2500×, and 5000× magnification respectively. D-F images of post-cycled material taken at 1250×, 2500×, and 5000× magnification respectively. G-I images of post-thermal runaway material taken at 1250×, 2500×, and 5000× magnification respectively. J-L images of cycled material post-thermal runaway taken at 1250×, 2500×, and 5000× magnification respectively.
